# The neutral condition in conflict tasks: On the violation of the midpoint assumption in reaction time trends

**DOI:** 10.1177/17470218231201476

**Published:** 2023-10-24

**Authors:** Parker Smith, Rolf Ulrich

**Affiliations:** Fachbereich Psychologie, Eberhard Karls Universität Tübingen, Tübingen, Germany

**Keywords:** Cognitive control, cognitive modelling, congruency effect, neutral condition

## Abstract

Although the relation between congruent and incongruent conditions in conflict tasks has been the primary focus of cognitive control studies, the neutral condition is often set as a baseline directly between the two conditions. However, empirical evidence suggests that the average neutral reaction time (RT) is not placed evenly between the two opposing conditions. This article set out to establish two things: First, to reinforce the informative nature of the neutral condition and second, to highlight how it can be useful for modelling. We explored how RT in the neutral condition of conflict tasks (Stroop, Flanker, and Simon Tasks) deviated from the predictions of current diffusion models. Current diffusion models of conflict tasks predict a neutral RT that is the average of the congruent and incongruent RT, called the midpoint assumption. To investigate this, we first conducted a cursory limited search that recorded the average RT’s of conflict tasks with neutral conditions. Upon finding evidence of a midpoint assumption violation which showed a larger disparity between average neutral and incongruent RT, we tested the previously mentioned conflict tasks with two different sets of stimuli to establish the robustness of the effect. The midpoint assumption violation is sometimes inconsistent with the prediction of diffusion models of conflict processing (e.g., the Diffusion Model of Conflict), suggesting possible elaborations of such models.

In many everyday situations, we may be distracted from an action goal by irrelevant information. For example, this is the case when we stop at an intersection and wait for a green light to turn left. If an additional traffic light for going straight turns green (the irrelevant information), this could distract us from our actual action goal, and we could end up in a dangerous traffic situation. Cognitive control is necessary in such cases to process goal-relevant information and to suppress goal-irrelevant information. It is therefore easy to see why the study of cognitive control is an important subject in cognitive psychology (Botvinick et al., 2001; J. D. Cohen, 2017; Norman & Shallice, 1986; Pashler, 1998; Posner & Snyder, 1975).

According to the most popular interpretation, cognitive control is necessary to distinguish between task-relevant and task-irrelevant information (J. D. Cohen, 2017; Kornblum et al., 1990). This is because, in many situations, task-irrelevant information is automatically processed, and thus interferes with the processing of task-relevant information (Posner & Snyder, 1975; W. Schneider & Shiffrin, 1977; Shiffrin & Schneider, 1977). The automatic process typically refers to actions done without directed cognitive effort and initiated by task-irrelevant information. However, controlled processing refers to goal-directed behaviour, such as navigation on dangerous terrain or careful, strategic actions. Thus, automatic processing happens constantly, with controlled processing adjusting or modulating the automatic process depending on the circumstance (van Veen & Carter, 2006). When this intervention happens, cognitive conflict occurs if the automatic response is opposed to the controlled response. This conflict is the basic effect addressed in studies of cognitive control, as it is an indicator of the different processing channels.

Various experimental tasks have been used to study conflict processing. Some of the most prominent tasks are the Eriksen flanker task (B. A. Eriksen & Eriksen, 1974; Miller & Schwarz, 2021; Servant & Logan, 2019), the Stroop task (Hedge et al., 2019; Steinhauser & Hübner, 2009; Stroop, 1935), and the Simon task (Chen et al., 2020; Mittelstädt & Miller, 2020; Simon, 1969; Simon & Rudell, 1967; Wühr & Heuer, 2018), each with different sources of conflict. In these tasks, participants are asked to respond to stimuli wherein two features of the stimuli can prompt opposing responses. For example, in the Eriksen flanker task (B. A. Eriksen & Eriksen, 1974), participants are shown a string of letters (e.g., HH K HH) and must respond to the central letter (i.e., K). In this example, the central letter might either be a “K” or an “H,” the task-relevant information, which is flanked by K’s or H’s, the task-irrelevant information. The task-relevant letters are mapped to the right and left response hands. In the congruent condition, the letter identity of the target letter and the flanking letters match, whereas in the incongruent condition, there is a mismatch. The root of this match or mismatch can be derived from the categorisation of the stimulus (as seen in Kornblum et al., 1990), where the letter string is possibly categorised as in favour of one response or the other. By altering the ease of discrimination between the flanking characters and the target (larger spacing between characters, more novel flankers), the influence of the flankers decreases, making varying congruency effect sizes. Thus, for the flanker task, conflict stems from a mismatch between a stimulus and its environment.

For the Stroop task, participants are shown the name of a colour in coloured font and are asked to respond to the colour of the font. For this task, the colour of the font is the target information, and the named colour is task-irrelevant information. Subsequently, responses are speeded if the named colour and font’s colour match, while they are inhibited if there is a mismatch. Drawing from Kornblum et al., 1990 again, the overlapping features of the stimuli cause the difference in the congruency effect. As the dimensional overlap of the features increases, such as the font of colour words becoming more similar to the semantic meaning, a larger congruency effect is seen. Thus, conflict in the Stroop Task is derived from mismatches between separate characteristics of a stimulus (such as semantic and visual information).

Finally, in the Simon task (Simon & Rudell, 1967), a red or blue dot is shown to the participants on the left or right side of the screen. Participants are asked to respond with the left or right hand to the blue or red stimulus, respectively. Therefore, the colour is the task-relevant stimulus dimension, whereas the dot’s location is task-irrelevant. In the congruent condition, the location of the dot and the response match, while in the incongruent condition, there is a mismatch. Although the location of the dot is task-irrelevant, it nevertheless affects response performance. So, for the Simon task, the response dimension is utilised to prompt the congruency effect. This creates a unique task, as the conflict does not solely rely on conflicting features in the stimulus dimension but across the stimulus-response set (for the Simon task, the location of the dot or sound and the hand which responds). Thus, it is the difference between the response and stimulus location that produces conflict.

## Elaborated diffusion models on conflict processing

Several stochastic models have been proposed to describe the underlying conflict process. Specifically, extensions of the standard diffusion model (Ratcliff, 1978; Ratcliff & Rouder, 1998; Smith, 2000) have been recently developed to address these conflict processes. This standard model assumes a noisy accumulation process towards one of two boundaries, each corresponding to a different response. This accumulation process reflects a Wiener Diffusion process with a linear drift towards one of these boundaries. As soon as the accumulation process hits one of the two barriers, a decision is made, and the corresponding response is activated. One conceptual disadvantage of this standard model is that it does not address the specific conflict mechanism. Therefore, extensions of the standard model have incorporated more specific assumptions about how response conflicts could arise. The most recent extensions are the *Dual-Stage Two-Phase* model (DSTP; Hübner et al., 2010), the *Shrinking Spotlight* model (SSP; White et al., 2011), and the *Diffusion Model of Conflict* (DMC; Ulrich et al., 2015).

**Figure 1.**
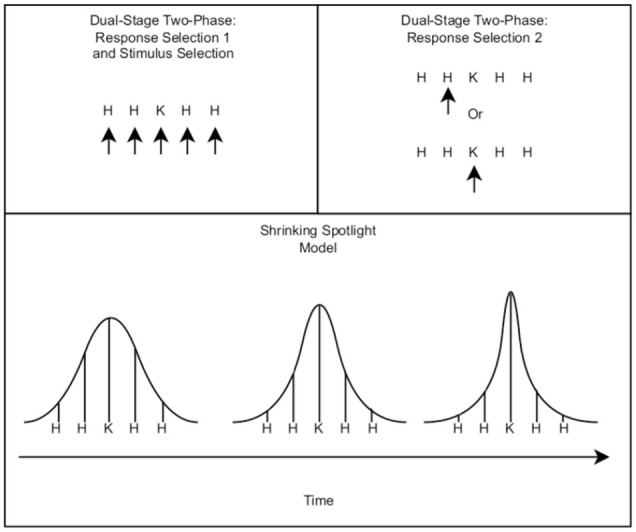
Above: An illustration of how the Dual-Stage Two-Phase operates conceptually. Top Left: The stimulus is shown, with the viewer considering all possible stimuli. As the process continues, the attention of the viewer (indicated by the arrows) may change between the flankers and the target; thus, the sum of the inputs forms the first Response Selection phase drift rate. When a stimulus is selected, Response Selection Phase 2 (Top Right) begins. If the stimulus selected is the flanking stimulus, the new drift rate will shift in favour of its associated response. Otherwise, the target is selected, with the drift rate accruing towards the correct response. Bottom: An illustration of the basic principles regarding the Shrinking Spotlight Model. In the image, the bell curves indicate the amount of visual attention given to each component of the stimulus over time. As time proceeds, the proportion of visual attention given to the outer and inner flankers shrinks, increasing the influence of the target stimulus.

DSTP takes inspiration from selective attention, with a focus on the idea of early and late selection (Kahneman & Treisman, 1984). It was specifically developed to model the cognitive processes underlying the flanker task. To mimic early selection, the first phase of response selection (RS1) in DSTP considers the participants as they first view both the flanking stimuli and the target stimulus (Figure 1). The model assumes a perceptual filtering process where each stimulus contributes to the information accumulation rate (Bundesen, 1990; Logan, 1996). This simulates the viewer’s initial exposure to the stimulus, taking both the target and flankers as equally viable sources of information. During this stage, the constant evidence rate of the target stimulus $\left(\mu_{tar}\right)$ and that of the flanking stimuli $\left(\mu_{fl}\right)$ are summed,

(1) $$\mathrm{Response}\;\;\mathrm{Selection}\;\;1:\;v(t)=\mu_{tar}\pm \mu_{fl}.$$

Although RS1 accumulates, a separate process called stimulus selection is simultaneously performed, wherein a target stimulus is selected as the proper target. This is modelled by utilising a standard diffusion model, with a drift towards the target stimulus. If RS1 reaches a response threshold before stimulus selection finalises, then the response corresponding to the threshold is performed. However, if the stimulus selection process finishes first, the second phase of response selection (RS2) begins upon its termination. The second response selection process follows the standard diffusion model framework, with an information accumulation rate corresponding to the constant evidence rate of the selected stimulus $\left(\mu_{rs2}\right)$ , until reaching a boundary and performing the associated response. This means that if the selected target is either the relevant stimulus or a congruent flanker, the accrual rate will bias towards the correct response threshold. However, if the selected stimulus is an incongruent flanker, the rate will bias negatively.

(2) $$\mathrm{Response}\;\;\mathrm{Selection}\;2:\;v(t)=\pm \mu_{rs2}.$$

**Figure 2.**
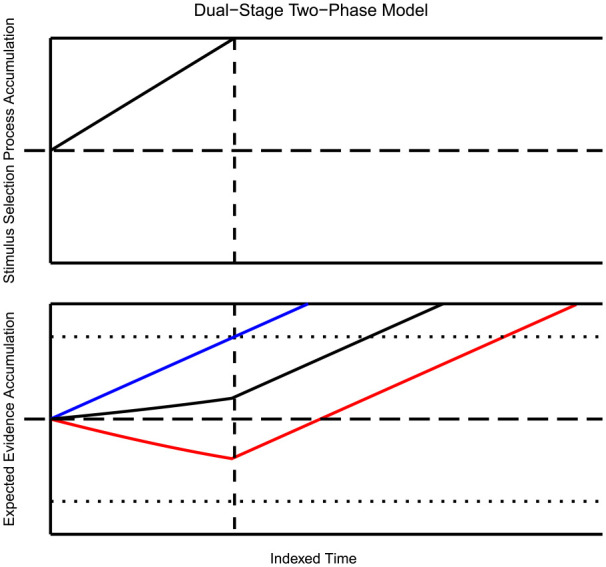
Dual-Stage Two-Phase Model: The top figure displays the deterministic Stimulus Selection process, which accumulates towards either the target or the flanker stimuli. The bottom figure shows the predicted drift of the response selection process as a function of time and for each experimental condition (congruent in blue, neutral in black, and incongruent in red). The horizontal line represents the decision threshold. The change in slope that occurs at the dotted line shows the transition between Response Selection Phase 1 and Response Selection Phase 2.

A notable part of this model is that it treats the selection of the target as a discrete process. Thus, the rates are fixed until a period is reached, only changing at the transition point from RS1 to RS2 . The resulting drift of the diffusion process implied by this model is depicted in Figure 2. Note that the intersection of the function v(t) with the horizontal line (i.e., the response threshold) corresponds to the point in time at which the decision process ends—for simplicity, a deterministic decision process is assumed in this and subsequent models. Overlaying the deterministic function v(t) with Brownian motion would only complicate the representation unnecessarily, which is why we prefer this simplification. Notably, this representation also assumes consistency in terms of the selection process, whereas in practice, the termination of this process may shift otherwise.

SSP was designed as an alternative to DSTP, as it simulates the viewer’s attention narrowing from the whole set of stimuli to just the target in a continuous fashion (C. W. Eriksen & Schultz, 1979; C. W. Eriksen & St James, 1986). The model approximates this by taking the Gaussian distribution and slicing its area into three sections: the outer edge, inner edge, and centre. The centre section is the information in favour of the target response area, while the outer and inner edges relate to the flankers’ influence on the information gained. These areas can be found by taking the appropriate integral over the area of interest. For the three area models discussed prior:

(3) $$\begin{array}{l} a\_\{outer\}(t)=\int_{-\infty }^{-1.5}\phi (x|0,\sigma_{a}(t))dx,\\ a\_\{inner\}(t)=\int_{-1.5}^{-0.5}\phi (x|0,\sigma_{a}(t))dx,\\ a\_\{target\}(t)=\int_{-0.5}^{0.5}\phi (x|0,\sigma_{a}(t))dx, \end{array}$$

where a_{outer}(t) is the influence of the outer flankers, a_{inner}(t) is the influence of the inner flankers, a_{target}(t) is the influence of the target, and $\sigma_{a}(t)$ is the function describing the area over time. The quantity $\sigma_{a}(t)$ is found by:

(4) $$\sigma_{a}(t)=\max(\sigma_{a}-r_{d}\cdot t,0).$$

Here, $\sigma_{a}$ refers to the width of the attention area, and $r_{d}$ is the rate at which attention area is narrowed. Intuitively, the model predicts that the longer a person looks at the stimulus, the smaller the variance becomes, decreasing the influence of the flankers and increasing the influence of the target (Figure 1). The accumulation of the decision process is obtained by summing up the influence of individual components over time. For example, if there are two flankers on each side of the target, the accumulation rate can be given as:

(5) $$\begin{array}{l} v(t)=2\cdot p_{outer}\cdot a_{outer}(t)+2\cdot p_{inner}\cdot a_{inner}(t)\\ \;\;\;\;\;\;\;\;\;\;\;\;+p_{target}\cdot a_{target}(t). \end{array}$$

**Figure 3.**
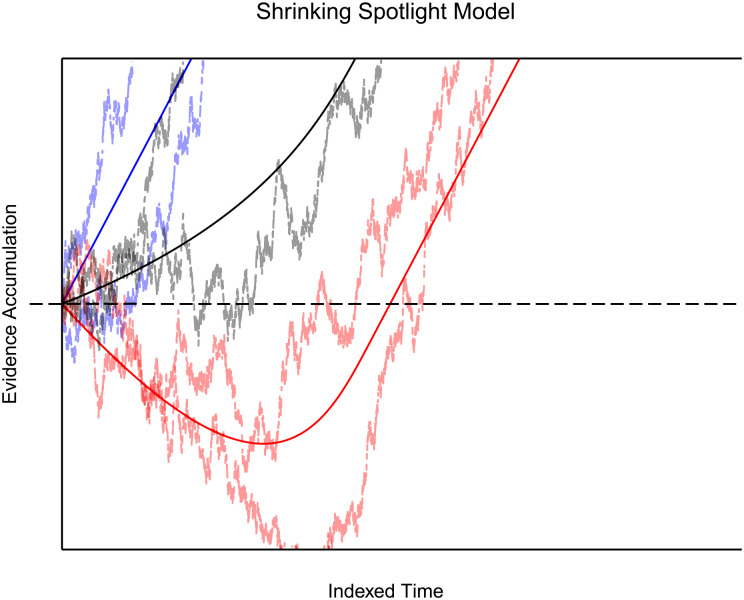
Shrinking Spotlight Model: The figure shows the deterministic time course of the diffusion process a continuous function of time for each experimental condition (congruent in blue, neutral in black, and incongruent in red). As before, the dotted, horizontal lines represent the decision thresholds. The gradual shift in the slope of the neutral and incongruent condition is derived from the gradual increase in attention given to the target stimuli rather than distractors. If stochastic noise is added, variability in the behaviour of these rates increases; however, the primary pattern of behaviour remains the same in the long run. The two runs of the noisy variant of the model (with a diffusion coefficient of σ=0.1 ) are included as the dashed lines following the drift paths.

The resulting function adjusts its curve as time progresses, increasing the rate of information gain in favour of the correct response. The predicted drift of the congruent condition, then, is consistently speeded and in favour of the correct response. In contrast, the incongruent condition drift is initially biased against the correct response threshold, then slowly increases as the proportion of selective attention attributed to the flanking stimuli diminishes. Thus, the a terms represent the selective attention-narrowing process, increasing the weight given to the central stimuli over time and lessening the weight granted to outer stimuli. Figure 3 depicts the resulting drift functions predicted by this model.

**Figure 4.**
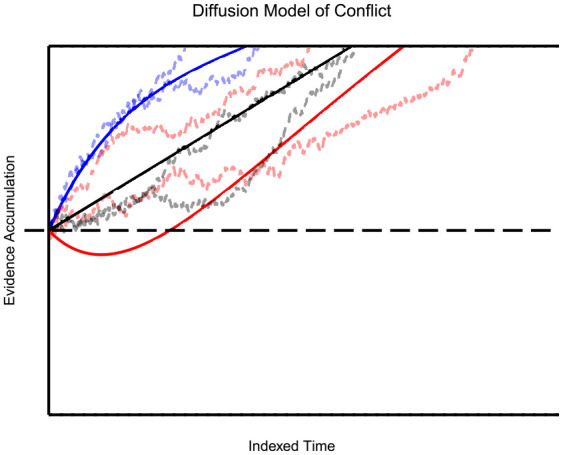
Diffusion Model of Conflict: With this figure, the predicted drift of the diffusion process is presented as a function of time for each experimental condition (congruent in blue, neutral in black, and incongruent in red). The congruent and incongruent time courses are a result of two overlapping processes: the controlled and automatic process. The neutral predicted drift rate utilises only the controlled process. The dotted, horizontal line represents the decision threshold, while the dashed line in the centre refers to the initial state of accrual. The two runs of the non-deterministic variant of the model (with a diffusion coefficient of σ=1 ) are included as the dashed lines following the drift paths.

DMC was developed to model the conflict tasks mentioned above, not just the flanker task. To accommodate the different types of tasks, the model focuses on simulating the automatic and controlled processes assumed in cognitive control. The controlled process is driven by task-relevant information (e.g., the central stimulus in the flanker task), while the automatic process is driven by the task-irrelevant information (e.g., the flankers). The controlled process assumes a constant drift rate µ_c_ towards the correct boundary. The automatic process generates a pulse-like activation that favours either the correct (congruent trials) or incorrect (incongruent trials) boundary. The drift rate of the automatic process is therefore time dependent, and scaled according to a gamma distribution, often set to the Erlang function. A major premise of this model is that the activation of the controlled process and automatic process are superimposed. Consequently, the drift rate as a function of t for this superimposed process is given by

(6) $$v(t)=A\cdot e^{-\frac{t}{\tau }}\cdot \left[\frac{t\cdot e}{(a-1)\cdot \tau }\right]\cdot \left[\frac{a-1}{t}-\frac{1}{\tau }\right]+\mu_{c},$$

where A is the amplitude of the pulse function, a is its shape parameter, and τ is its scale parameter. The result of these superimposed processes can be seen in Figure 4 as predicted time courses for the different conditions. DMC is able to both account for the time course of the congruency effect within the flanker and Stroop task (i.e., increasing effects with RT), as well as the Simon task (i.e., decreasing effects with RT).

## The neutral condition as a baseline condition

In most studies investigating the congruency effect in conflict tasks, only the congruent and incongruent conditions are used. This is surprising, as in other cognitive tasks (e.g., visual attention), a neutral condition acts as a baseline condition (cf., Posner & Snyder, 1975). Indeed, the absence of a neutral condition runs counter to some researcher recommendations (MacLeod, 1991). The reason that it is absent might be that it seems difficult to establish such a condition (Besner et al., 1997; Jonides & Mack, 1984; Neely, 1991).

Yet, a neutral condition may allow for additional insights into the underlying conflict mechanism (MacLeod, 1991; Parris, 2014). In such a condition, the stimulus should contain irrelevant information that does not promote either of the two response alternatives or prompts both responses to the same extent. For example, consider the colour Stroop task presenting the words RED or BLUE, where participants are asked to name the print colour of these words but ignore the word information. In a neutral condition, a non-word like XXXXX could be used as it does not present conflicting information (Schroeter et al., 2002). Similarly, for the Simon task, presenting a coloured dot in the centre of the screen should act as a neutral as well. Thus, the negation of any associated information is sufficient for the neutral task (Besner et al., 1997). The other possibility is to use a stimulus that triggers each of the two responses equally, such as a double-headed arrow for the arrow flanker task. Thus, the neutral task is obtained through either a lack of conflicting irrelevant stimuli or through the addition of irrelevant information that is both exciting and inhibiting at the same time. Due to the potential information loss done by neglecting a neutral condition, one is merely ignoring the elephant in the room and eventually hindering theoretical progress.

The most significant problem with neutral conditions is finding a stimulus that shares no similar features with either target stimulus, particularly in conflict tasks where the two information streams strongly interact. For example, the flanker task uses two fields of vision as competing information streams, so the primary mode of negation is to use a neutral that is unique from the target stimulus. However, it can be argued that similarities between visual stimuli may always exist; thus, a true neutral is difficult to obtain (Besner et al., 1997; Neely, 1991). Other issues can arise from the effects of third variable processes interfering with the neutrality condition, such as foveal processing with the Simon task (Mahani et al., 2019). For the Stroop task, different neutral stimuli can result in differing RT results depending on what kind of negation is used and its extent (Klein, 1964), implying certain neutral stimuli may be more or less similar to incongruent or congruent stimuli. Another danger is taking negation too far, substantially removing or altering the amount of information in a stimulus, as it could lead to speeded processing. For example, in the flanker task, one might assume presenting the target stimulus without any flankers would make for a good negation. Yet one could argue that, by removing the information that flanks the target, the stimulus is now rendered simpler. As the amount of information from the stimulus has decreased substantially, the task is fundamentally different from the other conditions, making speed comparisons between this “neutral” condition and the others a biased effort. This was already seen in the original flanker paper by B. A. Eriksen and Eriksen (1974), where responses to the single-letter alone condition were faster than in the congruent condition. Due to the spectrum of neutral possibilities, it is not always evident whether a neutral stimulus really functions as a proper baseline condition. This spectrum partially explains why the neutral condition is not often addressed in models of conflict tasks even though it could be informative, as we will argue below.

By consulting prominent RT models, different implications about the neutral condition’s behaviour are apparent. For example, drawing from established accumulation models (S. D. Brown et al., 2008; Grice et al., 1984) and the diffusion framework (Ratcliff, 1978; Ratcliff & Tuerlinckx, 2002), we can assume that the process of information accrual is roughly linear, with the neutral condition commonly lying between the congruent and incongruent processes. An implication of this property is that the difference between all of the average RTs, at any level of the criterion, will be a constant proportion. This draws from the idea of linearity, and assuming that the conditions begin accumulating at the same time. Specifically, this implication applies to DSTP and SSP (Figures 2 and 3). Moreover, these two models imply that the average RT of the neutral condition should be the average RT of the two opposing conditions. This property can be seen by plugging in a theoretical neutral condition into DSTP and SSP. In DSTP, the neutral condition stimulus selection would follow a standard diffusion model, while phase one of response selection accrues information from the flanking and target information. As the irrelevant information in the neutral condition is uninformative, one could expect a minimal accrual of information towards the correct response. Upon termination of the stimulus selection process, response selection phase two begins, leading most likely to the correct response as a selection of the neutral stimuli should not bias a response negatively. Admittedly, it is unknown if a selection of the neutral condition flanking stimuli is feasible, as that would theoretically lead to a non-response.

Similarly, for SSP, one can imagine that as the selection attention shrinks over time, no excitatory nor inhibitory information will be derived from the flanking stimuli. As with DSTP, the lack of congruent information still delays the accrual of information in comparison to the congruent condition but does not prompt the opposing response. Thus, to produce this effect in either model, one can set the flanking stimulus weights to zero, removing the flanking influence entirely, as seen in Figures 2 and 3. As this analysis shows, the inclusion of a neutral condition in assessing the empirical adequacy of these models appears to be a reasonable enterprise for further theoretical improvements.

If the neutral condition is the midpoint of the two standard conditions in conflict tasks, one should expect certain assumptions to hold. First, one would expect that across different conflict task studies, the difference between the conditions would be identical, that is,

(7) $$RT_{N}-RT_{C}=RT_{I}-RT_{N},$$

with $RT_{N}$ , $RT_{I}$ , and $RT_{C}$ being the neutral, incongruent, and congruent mean RT. Accordingly, it should be expected that the proportion of these differences would be identical; therefore, the ratio,

(8) $$R=\frac{RT_{N}-RT_{C}}{RT_{I}-RT_{N}}$$

should be equal to 1. Although some variability may alter this proportion, the overall trend should remain, provided the *midpoint assumption* (or *difference symmetry*) is maintained. If either the denominator or the numerator of the above proportion were to be larger or smaller than the other, we would state that there was violation of the midpoint assumption.

It is important to note that this proportion is only obtained from DSTP if the following assumptions hold. First, this assumes that the number of responses made in RS1 are relatively low. If there are a large number of correct responses from RS1 for both the congruent and neutral conditions, this could render R<1 . However, it is unlikely that the neutral condition would reach the response threshold of RS1 , as theoretically, the accumulation towards the correct response is quite low. Then, assuming that the proportion of neutral responses in RS1 is low, and that stimulus selection is accomplished at similar time intervals, R≈θ1 .

In regards to SSP, this prediction holds even when variability is introduced to the model. To test this, we consulted the article, White et al. (2011), for proper noise parameters and adjusted them for milliseconds. After running 2,000,000 trials, we found that the mean of these was R ≈ 0.972, leading us to find this result rather robust. The code used to produce these simulations is currently on Github, https://github.com/Parker−Smith−Uni−Tuebingen/NeutralConditionRSimulations.

Interestingly, DMC appears to violate the midpoint assumption. This can be seen in Figure 4, where the incongruent process arrives at the response threshold around 5 ms later than the neutral process, while the congruent process arrives 10 ms earlier than the neutral process. For a large number of parameter settings, DMC predicts a violation in the same direction, with R>1 .

This deviation from the other models is useful, however, as a growing amount of evidence runs counter to the midpoint assumption, as will become evident in the coming section. For example, Mahani et al. (2019) noted an odd occurrence when attempting to apply DMC and their own extension. Although their extension was able to account for a majority of patterns, it had difficulty handling asymmetrical RT differences in the data. According to them, the difference between the neutral and congruent conditions was smaller than that of the difference between the neutral and incongruent conditions, which the model was unable to predict reliably. Both of these observations clearly run counter to the midpoint assumption (symmetrical difference).

**Figure 5.**
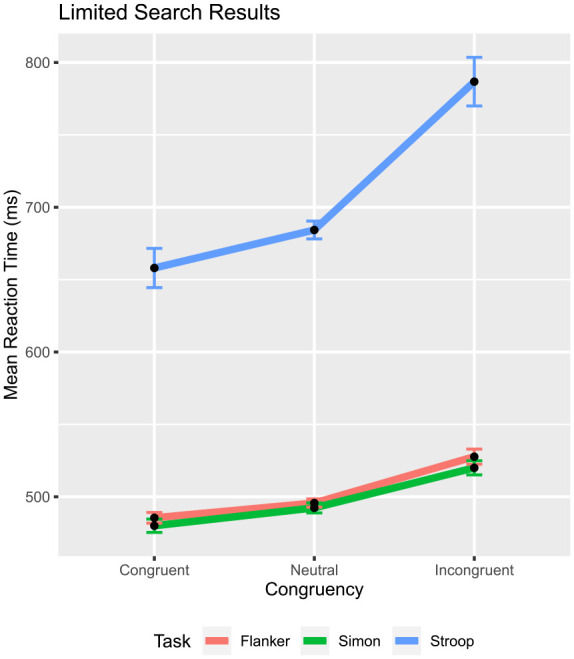
Result of limited search. Mean RT ± standard error as a function of congruency and conflict task. The error bar represents the standard error ± mean RT. The standard error was computed with Rmisc’s summary SE within function, which enables a comparison of the congruency effect within each task.

To assess the behaviour of the neutral condition in conflict tasks, a limited search of the literature was performed for the Eriksen flanker, Stroop, and Simon tasks, gathering a convenience sample of the three types. The workflow for selecting papers was as follows. The researchers used Google Scholar to search for papers due to its ability to search for specific words in an article. To search, the researchers entered each conflict task type with either “Neutral,” “Neutral Condition,” or “Neutral Stimuli” added on, and then they searched through the initial pages one by one (e.g., Flanker Task Neutral, Flanker Task Neutral Condition, Flanker Task Neutral Stimuli). Every paper link was then screened for the presence of a neutral condition in the paper. If a neutral condition (regardless of stimulus type) was found, the researchers would add it to a spreadsheet. After collecting the papers, the researchers then reviewed them for complications or issues that would affect the analysis (multiple types of “neutrals,” dual participants, only having neutrals and one type of test condition, etc.) Any paper that does not have a neutral condition was excluded. If the paper failed to report means for all conditions (graphically or in a table), it was excluded. Originally, the first five pages of Google Scholar were searched before another method was tried. However, further searching eventually required an exhaustive method of going through more than five pages. This process was repeated for the flanker, Simon, and Stroop tasks. The search was done from 1 January 2021 until 30 March 2021, then again from 12 February 2023 until 1 March 2023. It is notable that the second search specified papers prior to 1990 to gain a more historical perspective. Fifty-two articles (19 Stroop, 16 Flanker, 17 Simon) were collected, and in each case, the average RT per condition was recorded (Table 1). As is evident, the focus on this and all subsequent work in the paper is on the RTs of the given conflict tasks. The last column of Table 1 shows the outcome of Equation 8 for each article. It is readily apparent that the majority of articles (86.54%) recorded have an outcome R<1 , particularly for articles involving Stroop and flanker tasks (Figure 5). Interestingly, the opposite pattern is true for some of the Simon tasks, which have a sizable number of instances where R>1 .

**Table 1.** Limited literature search data. The above data are collected from various conflict task papers, all of which contain a neutral condition and present the RT’s for each condition. The papers vary in regard to stimuli type and response type. Here, *R* is equal to $\frac{RT_{N}-RT_{C}}{RT_{I}-RT_{N}}$ .

Papers	Conflict task	Congruent	Neutral	Incongruent	*R*
Atmaca et al. (2011)	Flanker	638	646	714	0.118
Sanders & Lamers (2002)	Flanker	493.5	486.8	511.3	–0.273
Davelaar & Stevens (2009)	Flanker	419.5	411.5	512.2	–0.079
Kopp et al. (1996)	Flanker	313.5	350.5	405	0.679
Salagovic & Leonard (2021)	Flanker	484	495.8	504.6	1.341
A. Cohen & Shoup (1993)	Flanker	477.7	496.5	514.3	1.056
Hsieh & Lin (2014)	Flanker	463.6	489.5	525.3	0.723
Richard et al. (2008)	Flanker	495	494.5	499.5	–0.100
Brion et al. (2018)	Flanker	477.8	469.7	485.7	–0.506
Dolk et al. (2014)	Flanker	343	350.5	362.5	0.625
Mordkoff & Chen (2021)	Flanker	443.9	457.4	484.7	0.495
Lawry (1980)	Flanker	788	805.5	831.5	0.673
Stoffels & van der Molen (1988)	Flanker	351.8	378.3	414.8	0.730
Shaffer & LaBerge (1979)	Flanker	572	587	618	0.484
Egeth (1977)	Flanker	411	412	448	0.028
Berry & Klein (1993)	Flanker	595.5	599.5	611.8	0.325
Ferraro et al. (2011)	Simon	346	351.5	370	0.297
Aisenberg et al. (2015)	Simon	504.1	518.9	544.6	0.576
Leuthold and Schröter (2006)	Simon	408.7	434.3	461.3	0.948
Burle et al. (2002)	Simon	393.5	347	421	–0.628
Folyi & Wentura (2015)	Simon	1019	1014.5	1033.5	–0.237
Yano (2011)	Simon	637.2	631.8	700.8	–0.078
Masaki et al. (2000)	Simon	307.5	351.6	427	0.585
Aisenberg & Henik (2012)	Simon	419.9	421.8	450.1	0.067
Salzer et al. (2014)	Simon	644.6	669	681.4	1.968
Mahani et al. (2019)	Simon	402.7	409.2	409.5	21.667
Hommel (1993)	Simon	397.9	423.7	442.9	1.351
Masaki et al. (2007)	Simon	406	412.5	421	0.765
Rubichi et al. (2000)	Simon	347	355.5	365	0.895
Hietanen & Pia (1995)	Simon	592	639	684	1.044
Stürmer et al. (2000)	Simon	445.3	462.3	486	0.718
Kunde (2003)	Simon	355.6	356.8	363.3	0.179
Rubichi & Pellicano (2004)	Simon	533	570.5	579	4.412
Chung-Fat-Yim et al. (2020)	Stroop	672	701	789	0.330
Schroeter et al. (2002)	Stroop	797.9	739	879.5	–0.419
West & Alain (1999)	Stroop	699.5	700	944	0.002
Salo et al. (2001)	Stroop	536.6	590.8	719.1	0.422
Roelofs et al. (2006)	Stroop	516	529	560	0.419
Kalanthroff et al. (2016)	Stroop	659.3	670.5	737.3	0.168
Compton et al. (2012)	Stroop	522.3	526.3	648.3	0.033
West & Alain (2000)	Stroop	639.5	642	754.5	0.022
Hasshim & Parris (2021)	Stroop	662.5	682	739.5	0.339
Kane & Engle (2003)	Stroop	632.2	682.3	852	0.295
Parris (2014)	Stroop	715	742	772	0.900
Yuan et al. (2021)	Stroop	632.1	660.2	726.7	0.423
Huang et al. (2020)	Stroop	608.7	638.5	700	0.485
Langton et al.(1996)	Stroop	681.3	679.5	703.8	–0.072
Lowe & Mitterer (1982)	Stroop	619.5	637.5	670.9	0.540
Stirling (1979)	Stroop	677.5	696.5	828.5	0.144
Jerger et al. (1988)	Stroop	963.3	1145.5	1459	0.581
Neill (1978)	Stroop	705	725	771	0.435
Kahneman & Chajczyk (1983)	Stroop	562.7	614	693.3	0.647

The results of this analysis are troubling, as DSTP and SSP both expect R=1 , thus are unable to account for this biasing effect given the assumptions prior. DMC is able to explain R≥1 , but has difficulties when addressing the most common bias of R<1 . DMC’s difficulty appears to stem from the linearity assumption of the controlled process, as the automatic process applies a pulse function in favour of either the correct or incorrect response. The result of this is that the congruent condition finishes quite quickly, while the neutral and incongruent conditions drag on. Such results can be seen in simulations of DMC, where only extreme parameter settings result in predictions of R<1 , as will be discussed in the General Discussion.

This violation of the midpoint assumption is rather consistent throughout the papers that were assessed. Papers with a diverse set of neutral conditions were included, making the skew towards a R<1 more peculiar. Although some researchers have stated that the R<1 pattern may be due to the specific neutrals used, one would expect that pattern to be averaged out by other studies. Thus, either a large number of studies in varied fields are utilising biased neutral condition stimuli, or this pattern may be something related to the controlled process. As the former seems quite unlikely, we are compelled to consider the latter.

One may wonder how stable the pattern of findings suggested by the limited search is. After all, a number of the papers had different types of target and irrelevant stimuli. To address this question, we conducted three pre-registered experiments, each employing a different conflict task (Experiment 1: Eriksen flanker task; Experiment 2: Stroop task; Experiment 3: Simon task). Each experiment included two sets of stimuli presented in separate blocks of each experiment. This was done to test the robustness of the effect, and to mimic the literature search’s variety of stimuli. Thus, we purposefully chose two distinct and separate kinds of stimuli to test the edge conditions of this phenomenon. In one set, the stimuli are composed of letters or words (linguistic set), while the other set contains shapes and symbols (symbolic set). With these two extreme versions of stimuli, we have hoped to cover the range of stimuli that we noticed in the publications that entered the limited search. The purpose of these experiments was to establish trends of the neutral condition rather than using the data to model. Thus, some experiments (such as the linguistic Stroop task) have more than two options. All experiments were performed online utilising Psychopy with Pavlovia (Peirce et al., 2019) and Prolific (2022), with SoSci Survey (Leiner, 2019) to collect participant consent. As these experiments were conducted online, the common concerns about participant motivation, controls, and variability in size are valid. For participant motivation, the use of strict cutoffs and the potential to not be compensated for bad data was used. As a number of papers have already addressed the concerns of online studies and found that controls and errors are within a reasonable range (Semmelmann & Weigelt, 2017), we also feel confident in the data from these experiments.

## Experiment 1—Eriksen Flanker Task

**Figure 6.**
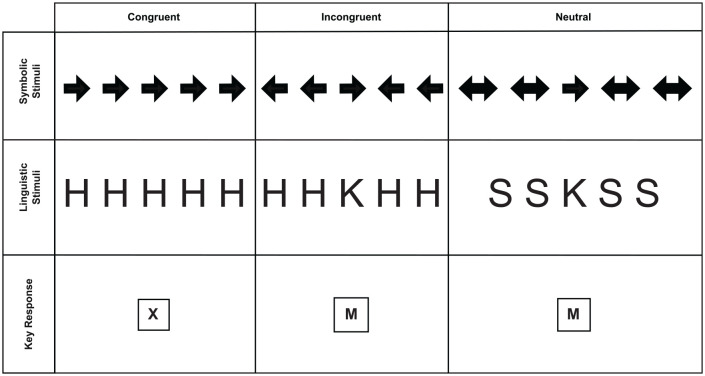
Stimuli Conditions for the Flanker Task: The examples of stimuli are presented in the upper rows, with an example of the response mapping appearing on the bottom row. Each stimulus set was presented in its own block.

Experiment 1 used two variants of the Flanker task, with each variant being a two-choice RT task. In the linguistic variant, the stimuli consisted of letter strings and participants were asked to respond to the target letter (H or K) in the middle of the string (Figure 6). The flankers could be congruent with the target stimulus to prompt the correct response, incongruent if they prompted the alternative response, or neutral if they prompted neither response alternative. In the symbolic variant, the target stimulus was an arrow pointing to the left or right response side (Figure 6). The flankers were also arrows whose direction may be congruent or incongruent with the response side. In the neutral condition, the flankers were double-headed arrows that likely primed both response alternatives, producing a net priming effect of zero in this condition. Each participant performed each alternative in a separate block of trials.

### Methods

#### Participants

Participants were required to have a fluent understanding of German and ownership of/access to a computer. We took into account the effect size expected from the limited search (Cohen’s d=0.68 ) as well as the observed effects during piloting when running the power analysis in G*Power (Faul et al., 2007). Considering both of these and specifying a power of 0.98 , we conservatively estimated a sample size of 50. Of the 50 that were recruited, seven were excluded due to a lack of correct responses (percent correct < 90%). The final total, then, was 43 participants (19 female, 22 male, 2 other, mean age =31.52 years, *SD* =12.29 years). Due to the strength of the results discovered after running the analysis, it was decided that no further participants were required. Each received a base rate of 13 dollars per hour, with the experiment lasting around 15 minutes in total.

#### Stimuli

The experiment was created within PsychoPy, using its experiment creator. Each stimulus set consisted of a string of five images shown in the centre of the screen: the central image as the target (H or K for the linguistic set, a left or right arrow for the symbolic set) with two flanking distractor images to the left and right. Sizing for these stimuli was done using the “height” scaling within PsychoPy, primarily due to the online experiment conditions. The scaling, therefore, is proportional to the screen size of the participant. Three different conditions (congruent, incongruent, and neutral) altered the image strings by changing the flanking images. For the congruent condition, the flanking images and target were the same, the incongruent condition had flanking images signalling the opposing response, and the neutral condition had flanking images signalling neither response (S for the linguistic set, a double-headed arrow for the symbolic set). Each image in the string was presented in a square that was 1/40th of the screen size. The horizontal string had a distance of 0.017 of the screen size between each image, meaning the whole string had a length of 0.168 of the screen size. For the linguistic set, white, Sans-serif characters were used. For the symbolic set, black arrow images were used to help participants differentiate the stimuli. The background for either stimulus set was a grey background, set to #808080.

#### Procedure

The flanker task experiment was created in PsychoPy and then uploaded to Prolific. Participants were recruited via a posting on Prolific, with a description of the task and requirements presented alongside it. Participant consent was collected upon starting the experiment via the SoSci Survey. Afterwards, participants clicked a link that brought them to the main experiment, wherein their age, gender, and Prolific identification number were requested. After providing the requested information screen, the participants were shown instructions for the first block of flanker stimuli. For the first 25 participants, the first set of instructions presented was for the letter stimuli, while the last 25 saw the symbolic stimuli instructions. For both tasks, the participants were tasked with responding to the central character of a stimulus string. To respond, they laid their left-hand index finger on the “X” key, and their right-hand index finger on the “M” key. For the letter flanker, participants had to respond to “H” with the “X” key, and to “K” with the “M” key. For the arrow flanker, participants responded to a left-facing arrow with the “X” key, and to a right-facing arrow with the “M” key. After reading through the instructions, the participants were instructed to proceed with 18 practice trials. Each trial consisted of a 500 ms presentation of a focus cross, followed by a 500 ms blank screen. Then, the stimuli were presented in the centre of the screen for a maximum of 2,500 ms. If the participants responded correctly, they were given no feedback. In case of an incorrect response, the message reading “falsche Taste” (Wrong Key) was displayed, and if they answered too quickly (less than 150 ms), the message reading “Zu schnell reagiert” (Reacted too quickly) was displayed. If no response occurred within the time frame, “Zu langsam reagiert” (Reacted too slowly) was shown. The stimuli sets were shown in a randomised order (as done by PsychoPy’s “loopType = random” setting), and balanced for an equal number of neutral, congruent, and incongruent trials.

After the practice trials, the participants pressed the “M” key to continue to the main session, 90 trials, for that stimuli block. After finishing these trials, the participants were presented with a screen displaying the set of instructions for the other stimuli (for the first 25 participants, the symbolic flanker instructions, for the latter 25, the letter flanker instructions), then proceeded with the experiment utilising the new instruction set. Thus, the first 25 participants were given the linguistic flanker task in the first block and then the symbolic flanker task in the second. This order was reversed for the last 25 participants in an attempt to counterbalance ordering effects for the different stimuli. The same number of practice trials and main trials were used again, as well as the experiment design. This is a pre-registered study, with the data and pre-registration available at https://osf.io/h3zwb/.

### Data analysis

**Figure 7.**
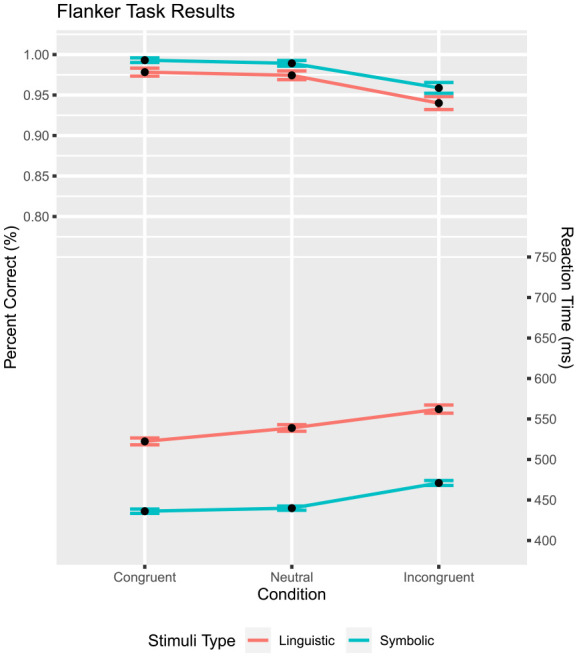
Flanker task: percent correct and Mean RT as a function of congruency and stimulus type. Mean RT is shown on the bottom half, percent correct is on the upper half. The error bar shows M±SE .

Trials with RTs less than 200 ms or greater than 2,000 ms were removed (linguistic stimuli: 0.2%, symbolic stimuli: 0.2%). These same cutoffs are used across all experiments. Only trials with correct responses were analysed (97.2%) for analysis on RTs. A repeated analysis of variance Congruency (congruent, neutral versus incongruent) × Stimuli type (symbolic versus linguistic) was performed on percentage correct (Figure 7). The expected accuracy differences for the flanker task were present across congruency conditions F(2,84)=27.36, p<.001, $\eta_{p}^{2}=.39$ . The accuracy was higher for symbolic stimuli than for linguistic stimuli, F(1,42)=18.94 , p<.001 , $\eta_{p}^{2}=.31.$ The interaction between the two factors was insignificant, F(2,84)=0.10, $\eta_{p}^{2}<.01.$ The same ANOVA was conducted for RT, which affirmed the standard flanker effect, F(2,84)=58.40, p<.001, $\eta_{p}^{2}=.58$ (Figure 7). Moreover and unsurprisingly, RTs were longer for linguistic than symbolic stimuli, F(1,42)=70.05 , p<.001 , $\eta_{p}^{2}=.63.$ Finally, the interaction between the two factors was insignificant, F(2,84)=2.64 , p=.077 , $\eta_{p}^{2}=.06$ .

However, upon visual inspection of the data, the symbolic stimuli revealed a possible violation of the midpoint assumption. This assumption was assessed by comparing the mean RT for the neutral condition to the average RT of the congruent and incongruent conditions with a two-sided, paired sample t-test. The difference was significant, with the results of a two-sided *t*-test being t(42)=6.05 , p<.001 for the symbolic set but not for the linguistic set, t(42)=0.648 , p=.521 . Thus, the *R* value for the symbolic set is significantly different from 1, while the *R* for the linguistic set is not significantly different from 1.

### Discussion

The violation of the midpoint assumption observed within the experiment is consistent with what is seen within the limited search (median *R* of 0.495). The ratio of RT differences for the symbolic set revealed R ≈ 0.122, coinciding with the limited search’s R<1 . Although the linguistic set displayed R ≈ 0.735, this outcome is not significantly different from 1. Due to this result pattern, there appears to be an effect of the type of stimuli on the severity of the midpoint assumption violation observed.

## Experiment 2—Stroop Task

**Figure 8.**
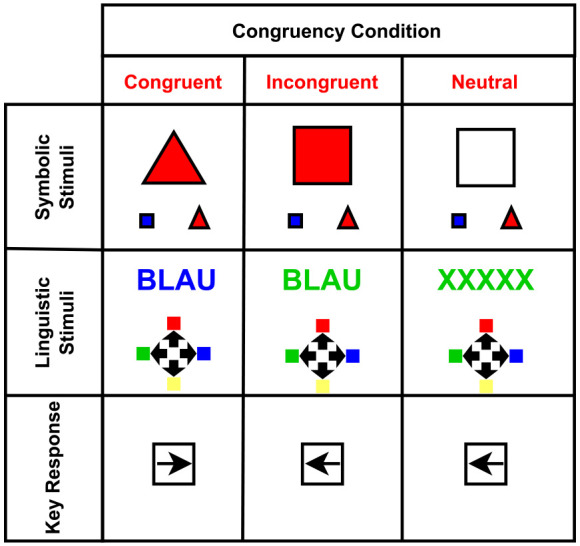
Examples of Stimuli Conditions for the Stroop Tasks: The stimuli examples are presented in the upper rows, with an example of the response mapping appearing on the bottom row. Each stimuli set was presented in its own block. The guide to the response in each stimulus example is shown on the screen consistently, to remind participants of key mappings for each response.

Experiment 2 used the same 2 stimuli formats as Experiment 1. In the linguistic variant, the stimuli consisted of colour words or a letter string (XXXXX), with participants asked to respond to the colour of the word or letter string (Figure 8). The colour of the word matched the name of the word in the congruent trials, incongruent trials were present if the colour of the ink was difference from the semantic meaning of the word, and neutral trials used a letter string as stimuli, prompting none of the responses. Participants had four response options (red, yellow, green, or blue) mapped to the arrow keys on the keyboard Figure 8. In the symbolic variant, the stimuli consisted of coloured shapes, where the participant responded to the shape (Figure 8). The shapes were presented with a guide at the bottom (a blue square and a red triangle), serving as a source of conflict. Congruent trials presented a shape coloured the same as the guide (blue square), while incongruent trials presented a shape coloured with the opposing response’s colour (red square). In the neutral condition, the shape was not coloured, negating the potential conflicting information. Each participant performed each alternative in a separate block of trials.

### Methods

#### Participants

The sample size was selected using the same procedure as Experiment 1. Participants were recruited from the same pool of participants as before with the same criterion for rejection participants due low accuracy. The specification of a QWERTY or QWERTZ keyboard was also added to the list of participant requirements to ensure a consistent key mapping. This specification was enacted after concerns over different keyboard layouts were expressed after the first experiment. This problem came about due to the online testing environment of our experiment. Thus, the use of arrow keys for responses was added, rather than alpha-numeric keys. Thus, the final total, then, was 50 participants (22 females, 26 males, 2 others, mean age = 26.31 years, *SD* = 8.77 years). As before, the base rate was 13 dollars per hour, with the experiment lasting around 15 minutes in total.

#### Stimuli

We once again used two stimuli sets, each involving a different variation of the Stroop task. The two variants of the task were the colour word Stroop Task and the Bivalent Shape Task (Mueller & Esposito, 2014). For the colour word Stroop task (Figure 8), we used four colour words (ROT, GELB, GRÜN, BLAU) along with a string of characters XXXXX. Each character in the word or string had a character height of 0.05 , or 1/20th of the screen size, and was coloured with a different ink (red, yellow, green, blue) where each ink was assigned a keyboard response. The participant was presented either a coloured word or string of X’s, with the congruent condition being a colour word with ink matching its meaning (ROT coloured red), the incongruent condition being a colour word with ink not matching its meaning (ROT coloured blue), and the neutral condition being a coloured string of X’s (XXXX). A reminder of the response mappings was presented at the bottom of the screen during the whole experiment (Figure 8), sized at (0.1,0.1) in terms of screen size (a square sized at 1/10th the screen size), and placed at the position (0,−0.25) on the screen. This stimulus set was used based on the traditional colour word Stroop task (Hedge et al., 2019; Steinhauser & Hübner, 2009). For the second variation of the Stroop task, the stimuli set was a coloured (blue, red, or white) shape (triangle, rectangle) placed in the centre of the screen (Figure 8). The size of each stimulus is 1/4 of the screen size area (sized at (0.25,0.25)) , and each was presented at the centre of the screen (position (0,0) ). On the bottom of the screen, a reminder of the response mappings was presented (sized at 1/10th of the screen size, each), with a red triangle on the right (position (0.15,−0.3)) and a blue rectangle on the left (position (−0.15,−0.3)) . This presentation creates the Stroop effect, as the congruent condition was the presentation of a shape with the same colour as the reminder (a red triangle), the incongruent condition being a shape coloured with the opposing response’s colour (a blue triangle), and the neutral condition being a white shape (a white triangle).

#### Procedure

Just like the previous experiment, the Stroop task experiment was uploaded to Pavlovia from PsychoPy before being posted on Prolific with a description of the task and requirements presented alongside it. Participant consent was collected upon starting the experiment. Afterwards, participants clicked a link that brought them to the main experiment, wherein their age, gender, and Prolific identification number were requested. Upon starting the experiment, the participants were first presented either the instructions for the linguistic or the symbolic task, depending on which Stroop variation they were enrolled in.

For the linguistic task, they were instructed to respond to the ink of the word/string on the screen, with each colour being mapped to an arrow key (Figure 8). The response options were up for red, right for blue, down for yellow, and left for green. They were to place their fingers on the arrow keys to respond quickly and accurately. As an example, if the word “Blau” (blue) appeared in a red font colour, the participant would respond with the up arrow key. For the symbolic task, they responded to the symbol with either the right arrow key for triangles or the left arrow key for rectangles. Thus, for a blue triangle, the participant would respond with the right arrow key.

The time intervals for the trials regarding fixation cross, blank space, and stimuli presentation as well as the feedback were the same as in Experiment 1. For randomisation, the same procedure was used as Experiment 1, with an equal number of neutral, congruent, and incongruent trials. After the practice trials (36 trials) were over, they then pressed the right arrow key to continue to the primary trials (120 for the linguistic Stroop, 118 for the symbolic Stroop). As in Experiment 1, the two variations of the Stroop task were performed in different blocks of trials.

### Data analysis

**Figure 9.**
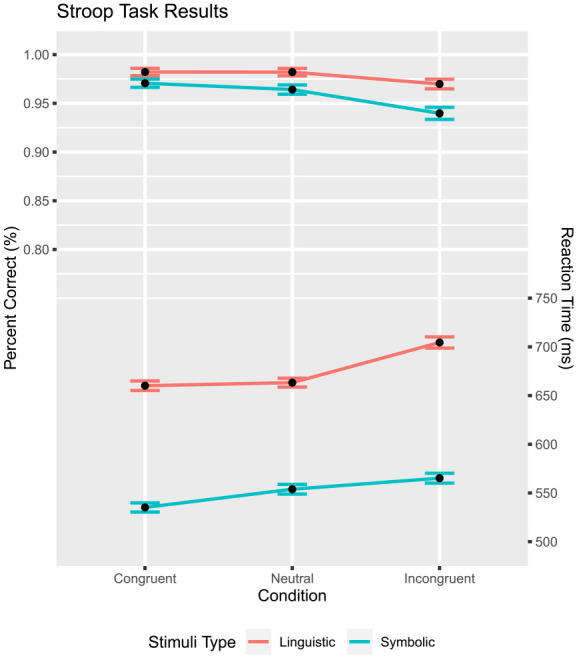
Stroop task: percent correct and mean RT as a function of congruency and stimulus type. Mean RT is shown on the bottom, percent correct is on upper half. The error bar shows M±SE .

The same data analysis as in Experiment 1 was performed. As before, trials with RTs less than 200 ms or greater than 2,000 ms were removed (Linguistic Stroop: 0.2%; Symbolic Stroop 0.3%). The analysis was performed on only correct responses (96.8%) from the data. Similarly to the flanker task, the expected accuracy differences were present across congruency conditions F(2,98)=16.06 , p<.001 , $\eta_{p}^{2}=.25$ (Figure 9). Although the linguistic stimuli had slightly higher accuracy, this only approached statistical significance, F(1,49)=3.98 , p=.052 , $\eta_{p}^{2}=.08$ . In line with previous assumptions, the interaction between the two factors was insignificant, F(2,98)=2.29 , p=.107 , $\eta_{p}^{2}<.04$ . The same ANOVA was conducted for RT as with the previous study, affirming the standard Stroop effect, F(2,98)=21.82 , p<.001 , $\eta_{p}^{2}=.31$ (Figure 9). RTs were longer for linguistic than symbolic stimuli, F(1,449)=75.94 , p<.001 , $\eta_{p}^{2}=.61$ . In contrast to the flanker experiment, the interaction between the two factors was significant, F(2,84)=6.46 , p=.002 , $\eta_{p}^{2}=.012$ . Upon visual inspection of the data, the linguistic, rather than the symbolic, stimuli seem to reveal a violation of the midpoint assumption. Again a t-test was used to test this assumption, which revealed a significant violation for the linguistic stimuli, t(49)=3.12 , p=.003 , but not for the symbolic ones, t(49)=−1.01 , p=.316 .

### Discussion

Similarly to Experiment 1, a violation of the midpoint assumption is observed. The stimuli set with the statistically significant violation was linguistic (R≈0.076) rather than symbolic (R≈2.01) . However, the significant violation appeared in the expected manner as the limited search (R≈0.330) .

## Experiment 3—Simon Task

**Figure 10.**
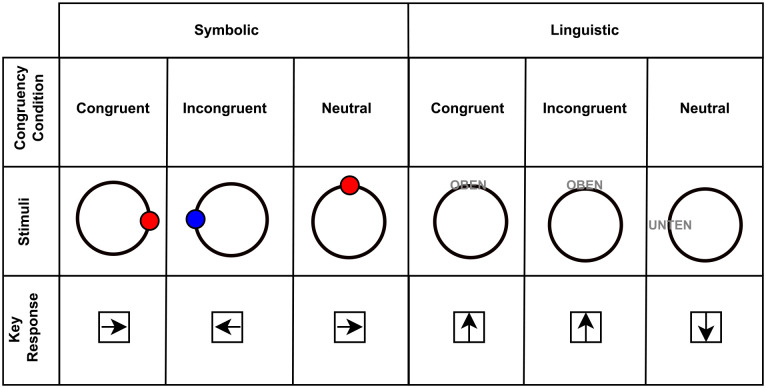
Stimuli Conditions for the Simon Tasks: The stimuli examples are presented in the middle row, with an example of the response mapping appearing in the bottom row. Each stimulus set was presented in its own block. It should be noted that the target stimulus could appear on the top, bottom, left edge, or right edge of the circle.

Experiment 3 used the same 2 stimuli format as the former experiments. For both sets, the stimuli appeared on top of a ring in the centre of the screen. This was done in an attempt to negate the foveal processing concern brought up in the literature (Mahani et al., 2019). In the linguistic variant, the stimuli consisted of two words, “oben” and “unten” (top and below), with participants asked to respond to the word’s meaning regardless of position (Figure 10). The position matched the semantic meaning in congruent trials, incongruent trials presented a mismatch, and neutral trials placed the stimuli at either side of the circle. For the symbolic variant, the stimuli consisted of coloured dots, where the participant responded to the colour of the dot Figure 10. The dots appeared on the right, left, top, or bottom of the ring. Congruent trials occurred when the response mapped to the colour matched the dot position (right arrow on red and left arrow on blue), while incongruent trials had a mismatch between dot placement and correct response (a blue dot presented on the left position). In the neutral condition, the dot was placed at the top or bottom the ring in an attempt to negate positional information. Each participant performed each alternative in a separate block of trials.

### Methods

#### Participants

Participants had the same requirements as Experiment 2. Similarly, with the Stroop Experiment, Prolific’s screening procedure allowed for an appropriate rejection and collection of replacement participants. The sample size of 50 was selected using the same procedure as Experiments 1 and 2. Thus, the final total was 50 participants (23 females, 26 males, 1 other, mean age = 25.99 years, *SD* = 7.79 years). Each received a base rate of 13 dollars per hour, with the experiment lasting around 15 minutes in total.

#### Stimuli

The third experiment follows the first and second experiment’s two stimulus paradigm, with two sets of stimuli presented in different blocks. The experiment was once again created in PsychoPy, but put online via Pavlovia. Across both sets of stimuli, a ring sized at 1/10th the screen area (presented in an image module sized at (0.1,0.1)) is presented in the centre of the screen (position (0,0)) . Noting that the fovea centralis covers an area of around 5.5º in the human visual field (Binder et al., 2009), and the largest typical screen size is around 723.00 × 477.52 mm (rendering a scaled stimulus within 5.17×3.42 degrees if participants are at least 800 mm away from the screen), we assume that the majority of participants will have the full ring within the range of foveal processing.

For the linguistic set, two words (“oben” or “unten,” meaning “above” or “down”) sized at a letter height of 0.02 (thus, 1/50th of the screen size each) in Arial font were presented to the participant at four possible locations on the ring in the centre of the screen (Figure 10). The position varies either in a directly horizontal or vertical position on the circle (0, π /2, π, 3 π/2). The conditions were congruent if the label was on the part of the circle which aligned with the proper response (example: “oben” at π / 2), incongruent if the label was on the inappropriate part of the circle (example: “oben” at π / 3). The neutral condition occurs if either label is on the π or 0 position. For the symbolic set, a dot (sized at (0.025,0.025)) varies in colour (blue or red) and position (0, π /2, π, 3 π/2) around the centre ring (Figure 10). The conditions were as follows: a congruent condition was if the response was aligned with the position of the dot (a red dot appearing at the 0 position, a blue dot appearing at the π position), an incongruent condition occurred when the response was opposite of the position of the dot (a red dot at the π position, a blue dot at the 0 position), and a neutral condition was when the dot was at a vertical position (π / 2 or 3 π / 2).

#### Procedure

As with the previous experiments, the task was presented as a Simon Task Experiment on Prolific, with a description of the task and requirements presented alongside it. Participant consent was collected upon starting the experiment. Afterwards, participants clicked a link that brought them to the main experiment, wherein their age, gender, and Prolific identification number were requested. Upon starting the experiment, the participants were presented either with the instruction set for the linguistic or the symbolic task, depending on which test set they enrolled in (semantic/symbolic for the first set and symbolic/semantic for the second set). For the linguistic task, participants responded to the word on the screen, with each meaning being mapped to an arrow key (“oben” with the up arrow key and “unten” with the down arrow key). For the symbolic task, they responded to the coloured dot with either the left arrow key for a blue dot or the right arrow key for a red dot. An example of the stimuli and proper responses are shown on the screen for clarity. Afterwards, they were presented with 24 practice trials, where trials had the same setup as the prior experiments. After the practice trials were over, participants then pressed either the right or up arrow key to continue to the 120 primary trials. As with the previous experiments, after they finished these trials, they were given the second set of instructions for the other stimuli. After reading, they continued the experiment with the new stimuli set and instructions for the same number of trials in both the practice and experiment sets.

### Data analysis

**Figure 11.**
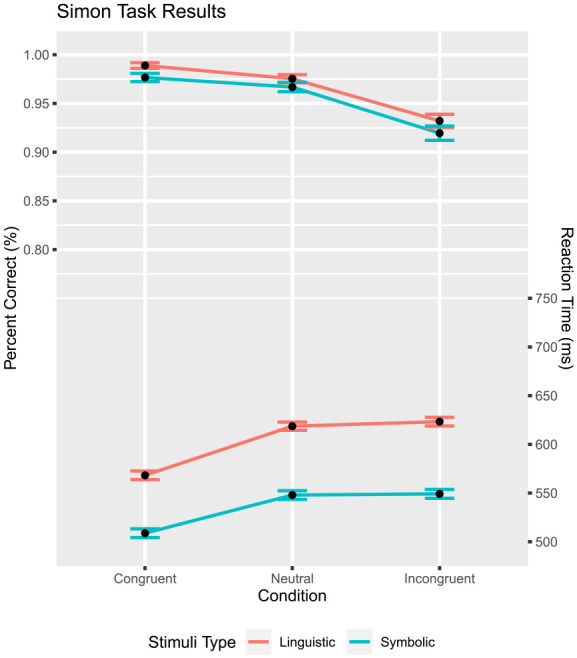
Simon task: percent correct and mean RT as a function of congruency and stimulus type. Mean RT is shown on the bottom, percent correct is on upper half. The error bar shows M±SE .

Slow trials were removed in the same manner as before (Linguistic Simon: 0.2%; Symbolic Simon 0.3%), and only trials with correct responses were analysed (96% of trials). Factor congruency produced again a significant effect on percentage correct, F(2,98)=66.25 , p<.001 , $\eta_{p}^{2}=.57$ (Figure 11). In addition, more correct responses were observed for linguistic than symbolic stimuli, F(1,49)=4.26 , p=.044 , $\eta_{p}^{2}=.08$ . Finally, the interaction between the two factors was insignificant, F(2,98)=0.12 , p=.862 , $\eta_{p}^{2}<.01$ , mirroring the results of the previous experiments. As expected, the ANOVA on RT produced a significant effect of congruency, F(2,98)=72.83 , p<.001 , $\eta_{p}^{2}=.60$ (Figure 11). Consistent with the previous two experiments, RTs were longer for linguistic than symbolic stimuli, F(1,49)=72.83 , p<.001 , $\eta_{p}^{2}=.60$ . The two factors, however, produced no significant interaction, F(2,98)=1.07 , p=.340 , $\eta_{p}^{2}=.02$ . Both stimuli sets suggest a violation of the midpoint assumption. In fact, a significant violation of the midpoint assumption was confirmed for the linguistic stimuli, t(49)=−5.66 , p<.001 , and for the symbolic set, t(49)=−2.82 , p<.01 .

### Discussion

Both stimuli sets violated the midpoint assumption, albeit in the opposite direction than was expected. The linguistic (R≈4.49) and symbolic (R≈15.3) sets displayed a neutral RT bias towards the incongruent condition. In contrast, the limited search showed the opposite pattern (R≈0.436) . Due to the efforts to remove foveal interference, it is surmised this did not play a role.

## General discussion

Experimental psychologists have proposed various conflict tasks (i.e., Stroop task, Eriksen flanker task, and Simon task) to study how irrelevant information affects goal-directed behaviour. These tasks usually focus on situations where the irrelevant information either conflicts with or facilitates the processing of relevant information. Due to this focus, situations where the irrelevant information neither conflicts nor facilitates are usually neglected. In this article, we have argued that the inclusion of such a situation, a neutral condition, can be informative about the mechanism underlying conflict processing. Specifically, the relation between the mean RT in the congruent, neutral, and incongruent conditions often violate the midpoint assumption; according to this assumption, the RT in the neutral condition should reflect the average of the congruent and incongruent condition RT. The prior empirical findings show that the midpoint assumption (symmetrical RT difference between the neutral and other conditions) is not given, as evidenced by the limited search and experimental results. As such, we believe that a more nuanced view of the neutral condition is warranted, as it should correspond to the controlled process.

Due to the unexpected behaviour, the neutral condition also serves as a good test for modelling and testing the veracity of said models, with a number of them only basing predictions off of the test conditions. For example, in the introduction, we have shown that the drift function (determining mean RT) of DSTP and SSP involves the midpoint assumption, whereas DMC does not. More specifically, DSTP and SSP predict an *R* approximately equal to one, whereas DMC predicts an *R* greater than one (i.e., a smaller difference in mean RT between the neutral and incongruent than between the congruent and neutral). As DSTP and SSP are solely based on the congruent and incongruent conditions, the midpoint assumption follows naturally. As shown further on in the General Discussion, one can incorporate the current findings of the neutral condition, however, and make model adjustments to account for these. We are particularly interested in the possibility of a nonlinear controlled process trajectory. Although these advances may seem incremental, these findings emphasise a negligence towards informative aspects of experimental design and show how said information can improve current models.

Our limited search of 52 studies revealed that it is common for *R* to be less than one regardless of task. Given this outcome, we investigated the robustness of this midpoint violation pattern, as some odd nuances were apparent. For example, the highest amount of negative *R* values appears to be associated with the Eriksen flanker task. Furthermore, previous studies have shown that mean RT for the neutral stimulus can vary widely depending on the type of stimuli used, at least when the Stroop task is concerned (Klein, 1964; Levin & Tzelgov, 2016). Drawing from these observations, we used different types of stimuli for all three conflict tasks to check the generality of the results. As stimuli could vary from levels of symbolic to linguistic in their meaning, we used clear examples of either case. We applied this experimental strategy to each conflict task and found that the results varied across tasks and stimuli types. Nevertheless, a clear effect of congruency was observed for each task and stimuli type. Moreover, there was also a consistent effect of stimulus type on RT, with longer RTs for linguistic rather than symbolic stimuli, presumably reflecting the additional process of reading (Logan, 1997).

However, stimulus type did not have a consistent pattern over how the midpoint assumption was violated across conflict tasks. For the Eriksen flanker task, the midpoint assumption was violated in the symbolic version but not in the linguistic version, while in the Stroop task, the opposite pattern was observed. Moreover, the Simon task exhibited an identical pattern of midpoint assumption violation, regardless of stimuli type. Curiously, the Simon task appeared resilient to stimuli type change, and responses are in line with DMC’s predictions. Even more curiously, the result of this experiment indicates an R larger than one, an exception established by the previous results and our limited search. However, it is still uncertain whether the patterns of *R* are linked to the way the stimulus is processed, the conflict process itself, or both.

When we altered the stimuli for each task, we theoretically stayed within the framework of the conflict task. However, it is possible that by altering the stimuli, the way the conflict task was interacted with was also altered. For example, changing the letters to arrow may have unintentionally prompted the correct response, as having a single arrow pointing in the correct direction may have caused the participant to disregard the other head. Then, in the linguistic Simon task, one could argue that it is more of a spatial Stroop task. If that is the case, then the new neutral bias present there is clearly different from the expected neutral behaviour given our limited search of the literature. Thus, the stimuli type seems to act without a specific pattern on neutral condition bias, at least from our current investigations. This inconsistent effect of stimuli type on the midpoint violation indicates that the type of conflict may play a role. That is, the type of conflict found in each task may affect the way participants respond to different neutrals.

As discussed in the introduction, each conflict task relies on a particular type of conflict to generate the effect. For the flanker task, conflict is generated by the disparity between a stimulus characteristic and its environment. From the data we collected, it appears that the facilitation effect (difference between the congruent and neutral RT) can vary in intensity, resulting in R≤ 1. If one considers the stimulus and its environment to be a singular stimulus set, it is not surprising that the Stroop task also follows a similar pattern. After all, the Stroop task relies on differences in stimulus attributes for conflict, so if the environment and the target in the flanker task are considered different attributes, the two tasks are quite alike. However, in the Simon task, the source of conflict comes from the disparity between the stimulus and the response. It may be the case that the relative positioning of the Simon task stimuli could result in participants treating any stimulus that is not distinctly on the response side as incongruent. For example, if a blue dot assigned the response “Left” appears on the left, the response is congruent, and if it appears on the right side, it is incongruent. However, if the dot appears in the centre, it is relative to the right of the congruent response placement, possibly adding an incongruous element. Thus, depending on the type of conflict, one could imagine different tendencies for the controlled process. However, it is important to note these cases are still based on speculation, and more research is needed.

With the potential variation of neutral condition behaviour based on stimuli or conflict, readers may even question the concept of a neutral condition (Jonides & Mack, 1984). For example, one might assume that the neutral stimulus is sometimes associated with the correct response and thus propose that this should result in speeded RTs preferring the congruent condition (i.e., producing R<1 ). However, in other cases, the neutral stimulus may be associated with the incorrect response, proposing a preference for the incongruent condition with delayed RTs (i.e., producing R>1 ). Finally, if the neutral condition appears to prime neither or both responses, one might conclude it is unbiased with an R=1 .

Although this latter assumption seems reasonable, it does not always line up with the data. For example, in Experiment 1, we used two types of neutrality, with the symbolic priming of both responses and the linguistic priming of neither response. Although this assumption implies both should result in the R=1 , this was not the case (e.g., the symbolic stimuli produced R<1 , the linguistic stimuli produced an R=1 ). Another argument comes from the limited search, where the average *R* across all tasks was R<1 . It seems unlikely that, given the framework before, the majority of the studies would only favour the congruent condition rather than balancing it out across studies.

**Figure 12.**
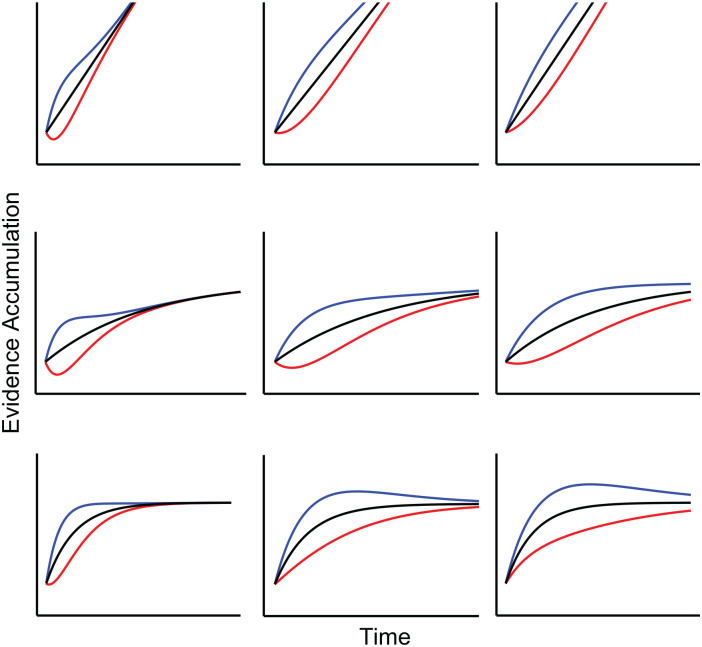
Nonlinear Extension of DMC: The figure shows various possible time courses for the nonlinear extension. Across the images, τ changes from 50, 100, and 150 going left to right. The top row displays a setting for the standard model of DMC, where c is set to close to 0. Going from top to bottom, c is increased from *c* ≈ 0, c=0.004 , to c=0.013 .

Perhaps, then, we have trapped ourselves with the midpoint assumption, while the true mechanism appears to act quite differently than a simple associated priming of responses with the correct or incorrect response. As outlined in the introduction, diffusion models generally assume that the accumulation of task-relevant information involves a linear accrual of information. A plausible alternative is that information accrual involves a diminishing rate of return, resulting in a concave accumulation function (Grice et al., 1982, 1984), allowing for different expectations of *R*. For example, DMC involves the standard assumption of diffusion models that the accumulation of task-relevant information is linear, implying a constant rate of information. However, DMC primarily expects R≥1 , rather than allowing for, the more commonly observed R<1 . If one applies the diminishing return principle to information accumulation, the drift rate of the controlled process may be modelled as an exponential decay function, that is, $\mu_{c}(t)=\mu_{c}\cdot \exp(-c\cdot t)$ , which would correspond to a concave accrual function $E[X_{c}(t)]=\frac{\mu_{c}}{c}\cdot [1-\exp(-c\cdot t)]$ for DMC’s controlled process.^1^ Note that if c approaches zero, the accumulation function would be linear, i.e., $E[X_{c}(t)]=\mu_{c}\cdot t$ , that is, this elaboration still includes the original DMC version. An example of the modified DMC model is depicted in Figure 12, where differing values of τ and curvature parameter c result in different levels of *R*. As this figure demonstrates, a concave accrual function of the controlled process may accommodate the diversity of the observed *R* in the literature without requiring additional assumptions.

**Figure 13.**
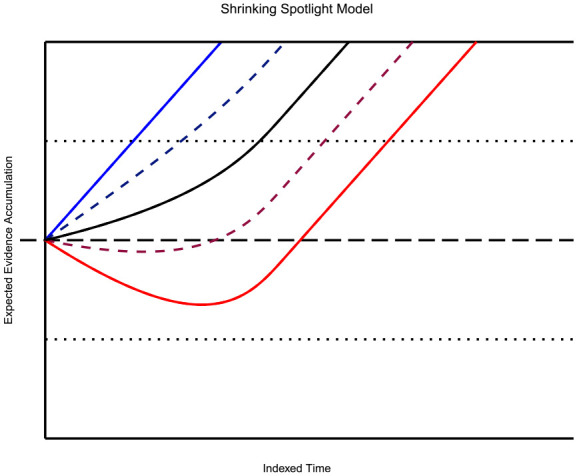
The above graph shows how the addition of a continuous parameter attached to the congruency parameter can allow for a flexible neutral condition RT estimate. By shifting the placement of the theoretically controlled process, different biases can be simulated. For positive values of b , the neutral biases towards the congruent condition RT, and for negative, the neutral is biased towards the incongruent condition RT.

For SSP and DSTP, midpoint assumption violations stemming from stimulus processing are presumably the primary concern, as they both relate to a single type of conflict (i.e., the flanker effect). Considering that, for the flanker task, R is mostly less than one, an extension of these two models need only account for a potential bias towards the congruent RT. One interpretation of this midpoint violation is that the impact of the automatic process is modulated by congruency condition (Evans & Servant, 2022). Another possibility, stemming from the previous extension of DMC, assumes that the controlled process is biased during its time course. For example, such a bias may arise when flankers and the targets share features (e.g., B. A. Eriksen & Eriksen, 1974). To account for the mentioned bias, a parameter can be added to DSTP and SSP. For SSP, it is usually assumed that the flanking information has either a positive or negative effect on target selection, depending on congruency. If this is made into a parameter, a gradient of congruency is possible. Accordingly, Equation 5 can be modified for the neutral condition as such

(9) $$\begin{array}{l} v(t)=b\cdot [2\cdot p_{outer}\cdot a_{outer}(t)+2\cdot p_{inner}\cdot a_{inner}(t)]\\ \;\;\;\;\;\;\;\;\;\;\;\;\;+p_{target}\cdot a_{target}(t) \end{array}$$

with the bias b parameter being bounded as −1<b<1 . More specifically, b>0 implies R<1 (i.e., the neutral RT is more similar to RT in the congruent condition), whereas b<0 implies R>1 (i.e., the neutral RT is more similar to the RT in the incongruent condition). These implications are depicted in Figure 13. A similar modification applies to DSTP, especially to Phase 1 processing in Equation 1.

In this article, we have focused on the predictions of elaborated diffusion models and how a neutral condition could expand the database for these models to evaluate them even better. For this reason, we have excluded models that do not assume a diffusion process. For example, Miller and Schwarz (2021) have suggested an activation suppression model within the discrete information processing framework. The model assumes a race of two processes: A and B. Process A suppresses task-irrelevant information, which takes time to be successful. Process B operates on task-relevant information. If process A finishes before B, subsequent information processing is not affected by task-irrelevant information. By contrast, if B finishes before A, subsequent information processing interferes with task-irrelevant information. Thus, the congruency effect is crucially dependent on the speed of process A, for if A is always faster than B, no congruency effect will emerge. This model is agnostic with regard to the neutral condition. However, it would assume that the neutral condition is somewhere in between $RT_{I}$ and $RT_{C}$ due to its logic. Consequently, the neutral condition would presumably not enable a better evaluation of this discrete conflict task model.

In summary, the origin of the different R values observed in the literature can be explained well, at least qualitatively, on the basis of diffusion models for conflict tasks. This, of course, provides clues as to how to further develop such models to also include neutral conditions in their quantitative assessment. Unfortunately, however, as we have seen, this also requires that at least one parameter be added to these models, which further complicates the estimation of model parameters (White et al., 2018) but is probably quite feasible, as other elaborations of these models show (Evans & Servant, 2022). As a first step, however, we believe it important to assess whether certain phenomena would be conceptually consistent (in the sense of a proof of concept) if these models are extended in a plausible way. Consequently, considering the neutral condition, rather than neglecting it, may highlight problematic assumptions of the current models for conflict tasks.

## Notes

1. The expectation of $X_{c}(t)$ is obtained by integrating µ_c_ (*t*), that is, $E[X_{c}(t)]=\int_{0}^{t}\mu_{c}(x)\;dx$ .
